# Sound Practice—improving occupational health and safety for professional orchestral musicians in Australia

**DOI:** 10.3389/fpsyg.2014.00973

**Published:** 2014-09-09

**Authors:** Bronwen J. Ackermann, Dianna T. Kenny, Ian O'Brien, Tim R. Driscoll

**Affiliations:** ^1^School of Medical Sciences, Sydney Medical School, University of SydneySydney, NSW, Australia; ^2^Faculty of Arts and Social Sciences, University of SydneySydney, NSW, Australia; ^3^School of Public Health, Sydney Medical School, University of SydneySydney, NSW, Australia

**Keywords:** professional orchestral musicians, music performance anxiety, performance-related musculoskeletal disorders, workplace health and safety, noise-induced hearing loss

## Abstract

The Sound Practice Project is a 5-year study involving baseline evaluation, development, and implementation of musician-specific work health and safety initiatives. A cross-sectional population physical and psychological survey and physical assessment were conducted at the same time, with an auditory health assessment conducted later. The results were used to guide the development of a series of targeted interventions, encompassing physical, psychological, and auditory health components. This paper provides an overview of the project but focuses on the health findings arising from the cross-sectional survey. Three hundred and seventy-seven musicians from the eight professional symphony orchestras in Australia took part in the cross-sectional study (about 70% of eligible musicians). Eighty-four percent (84%) of musicians reported past performance-related musculoskeletal disorder (PRMD) episodes; 50% were suffering a current PRMD. Of the 63% who returned hearing surveys, 43% believed they had hearing loss, and 64% used earplugs at least intermittently. Noise exposure was found to be high in private practice, although awareness of risk and earplug use in this environment was lower than in orchestral settings. Improved strategic approaches, acoustic screens and recently developed active earplugs were found to provide effective new options for hearing protection. With respect to psychosocial screening, female musicians reported significantly more trait anxiety, music performance anxiety, social anxiety, and other forms of anxiety and depression than male musicians. The youngest musicians were significantly more anxious compared with the oldest musicians. Thirty-three percent (33%) of musicians may meet criteria for a diagnosis of social phobia; 32% returned a positive depression screen and 22% for post-traumatic stress disorder (PTSD). PRMDs and trigger point discomfort levels were strongly associated with increasing severity of psychological issues such as depression and music performance anxiety.

## Introduction

**Figure 1:**
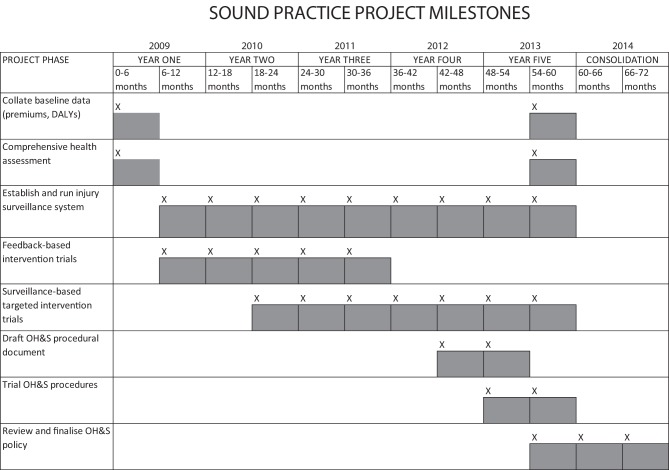
**Sound Practice Project—initial project timetable**.

Professional orchestral musicians are required to practice highly complex actions for many hours on a daily basis to develop the elite skills that enable them to perform classical masterpieces in prestigious venues under the scrutiny of large and mostly discerning audiences. To be able to achieve this kind of success in this competitive industry requires optimal physical and psychological functioning of the musician. Such a high-pressured workplace often requires musicians to push their physiological boundaries to the limit, thereby making them highly vulnerable to injury. Since the 1980s, literature consistently reports significant health concerns, including high rates of performance-related injuries, psychological health issues such as music performance anxiety, and reports of noise-induced hearing loss (NIHL). In 2009 a team of University of Sydney researchers were funded to undertake an occupational health project, the *Sound Practice* project, which comprehensively investigated the health of orchestral musicians across Australia. An initial baseline cross-sectional survey of the physical and psychological health of the population, and a physical assessment, were conducted at the beginning of the study, with an auditory health assessment conducted later. The results of these assessments were used to guide the development of a series of targeted interventions, encompassing physical, psychological, and auditory health components (Figure 1). This paper provides an overview of the background to the project and presents a summary of the main health findings of the cross-sectional component of the study which underpinned the interventions that were later piloted. These have been reported in more detail elsewhere (Ackermann et al., 2012; Driscoll and Ackermann, 2012; Kenny et al., 2012; O'Brien et al., 2012; Kenny and Ackermann, 2013).

### Physical aspects and injury

Literature documenting the health risks and high rates of injury faced by musicians internationally has been steadily building over the last few decades, and yet the statistics of players suffering performance-related injuries has remained essentially unchanged (Fry, 1986; Fishbein et al., 1988; Leaver et al., 2011; Paarup et al., 2011; Ackermann et al., 2012). Most reports indicate that approximately 80% of players will experience an injury that affects their ability to play their instrument, and that these performance-related injuries are even common amongst the school aged talented students (Ranelli et al., 2011).

While playing related overuse or misuse have been commonly cited as the generic cause of injuries occurring to this population (Fry, 1986; Meinke, 1998), it is becoming increasing clear that a multitude of risk factors are involved in injury causation (Manchester, 2007; Brandfonbrener, 2010). Lack of prospective injury surveillance data hampers attempts to infer injury causality from exposure to previously identified risk factors (Ackermann and Adams, 2004; Brandfonbrener, 2010).

In an orchestra review published in Australia in 2005, the author noted that orchestral occupational health and safety practices could be significantly improved world-wide, and recommended that funding be allocated to address this issue in the Australian orchestras (Strong, 2005). The *Sound Practice* project answered this call to perform a longitudinal occupational health study of the eight main professional orchestras in Australia over a 5-year period. The project comprehensively investigates the health and well-being of professional orchestral musicians and trials various health-focused programs and interventions.

### Psychological aspects

International surveys of professional orchestral musicians have always reported high rates of music performance anxiety (Fishbein et al., 1988; James, 1997, 1998) but the studies are old and did not use standardized assessment tools, making comparisons among studies impossible. Thus, to date, the mental health of professional musicians has not been well-understood; hence, their mental health needs are often poorly managed. Psychosocial health including health behaviors such as substance and alcohol use have not been systematically studied and this needed to occur in order that health prevention programs target the appropriate health behaviors for the appropriate subgroups if these be identified.

Despite relationships between pain and stress, and anxiety and depression having been extensively examined in different pain populations, few studies have assessed these relationships in populations of professional orchestral musicians. The results of our study demonstrated an interactive relationship between psychological factors, such as music performance anxiety and depression and playing-related musculoskeletal symptoms. It is also possible that performance-related musculoskeletal disorders have an impact on psychological wellbeing. Evidence of an interactive impact between hearing and psychological health, with self-reported hearing problems associated with a perceived poorer psychosocial environment, mental health symptoms and stress has been observed in this population (Hasson et al., 2009).

While more work is needed, particularly the conduct of longitudinal studies that track the young musician from entry into an orchestra over a number of years to assess causal mechanisms for physical and mental health concerns, the current research has highlighted complex relationships between psychological health and physical factors that would be profitable to explore further with a view to developing effective management and treatments for the impaired musician. It is possible that may have some kind of similar complex relationship.

### Noise

It is by now well-established that, while playing in the orchestra, orchestral musicians may be exposed to levels of sound that risk permanent damage to their hearing. There have been many sound level surveys of orchestras published in the literature, most in the last 40 years. These studies have used a variety of methods and interpretations, but many have reached the conclusion that the risk of NIHL in the orchestra is highly variable dependent upon instrument type, venue, repertoire, and duration of exposure, and that the greatest exposure is generally from a musicians' own instrument (Axelsson and Lindgren, 1981; Jansson and Karlsson, 1983; Lee et al., 2005; O'Brien et al., 2008; Schmidt et al., 2011).

Audiological investigations have produced conflicting results, with some finding limited evidence of hearing loss (Jansson and Karlsson, 1983; Obeling and Poulsen, 1999; Kähäri et al., 2001), although many more have found that professional orchestral musicians exhibit greater incidence of noise-related hearing pathologies (including permanent threshold shift, tinnitus, and hypercusis) than the general population (Emmerich et al., 2008; Jansen et al., 2009; Pawlaczyk-Łuszczyńska et al., 2010; Toppila et al., 2011). Evidence of increased incidence of hearing loss amongst the higher exposed musicians in this population continues to emerge (Wilson et al., 2013).

As Australia's orchestras all had some form of hearing conservation in place at the commencement of the *Sound Practice* study, the current strategies and approaches used by orchestral managements were investigated. In addition, the hearing conservation habits and attitudes of the musicians were examined. This information was used as the basis of recommendations to improve noise control measures.

## Materials and methods

### Participants

Australia has eight full-time professional symphonic and pit orchestras located in each of the capital cities of Australia. Their musicians represent the country's most elite orchestral musician population. With few elite positions available for a relatively large number of aspiring musicians, intense competition exists to acquire a position in one of these professional orchestras. All orchestral musicians employed by these eight major state orchestras were invited to participate in the study. This included permanent and casual musicians.

### Physical

A baseline questionnaire was developed that incorporated pain and injury questions refined from a variety of sources including previous musicians' health research surveys developed by one of the authors (BA); existing commonly used survey tools; and additional musician-specific questions recommended by leading international musicians' medicine experts. This was completed by participants at the beginning of the study.

A team of trained physical therapists conducted a baseline physical examination on each participating musician. This protocol was developed by one of the authors (BA) and was based on current best-evidence guides. A protocol was developed using a range of procedures to test strength, range of motion, neural sensitivity, and fine motor control (Ackermann and Driscoll, 2010). All testers attended a protocol-training day and were given a detailed booklet to optimize consistency. Inter-rater reliability tests were conducted. The testing took place at each orchestra location and took approximately 1 h per musician (in addition to the 1 h for the physical and psychological health surveys).

### Psychological

#### Assessment of psychological characteristics of professional orchestral musicians

This cross-sectional population survey of the musicians (*n* = 377) consisted of a detailed questionnaire, administration of standardized psychological tests and a physical examination. The aim was to assess the prevalence and reported causes of music performance anxiety within this population, and to provide some normative data on a range of psychological screening tests for professional musicians.

A range of validated psychosocial measures were included. These were: *Kenny Music Performance Anxiety Inventory (Revised)* (K-MPAI) (Kenny, 2009)^1^; *Trait questionnaire of the State-Trait Anxiety Inventory* (STAI-T) (Spielberger et al., 1983); *Social Phobia Inventory* (SPIN) (Connor et al., 2000); *PRIME-MD Patient Health Questionnaire* [PRIME-MD PHQ (Spitzer et al., 1999)]; *Anxiety and Depression Detector* [*ADD* (Means-Christensen et al., 2006)]; *Core Self Evaluation Scale* [CSE (Judge et al., 2003)]; and *Alcohol Use Disorders Identification Kit* [AUDIT (Babor et al., 2001)]. Detailed descriptions of these scales can be found in Kenny et al. (2012).

In the absence of suitable instruments, a number of checklists, questionnaires, and scales were developed specifically for this study. These included:

- Performance anxiety in different performance settings rating scale (Kenny, 2011).
- Workplace satisfaction scale for musicians (Kenny, 2009).
- Workplace environment scale for musicians (Kenny, 2009).
- Causes of Music Performance Anxiety Checklist (Kenny, 2011).
- Self-management of music performance anxiety rating scale (Kenny, 2011).

#### In-depth psychological interviews

A series of in-depth interviews were conducted with a sub-group of orchestral musicians who self-identified as suffering from extremely severe levels of MPA. All 20 interviews were audio-taped and transcribed in full. The aim of the analysis was to extract common themes in the transcripts that would provide a nuanced insight into the psychological lives of orchestral musicians in order to gain further insight into their psychodynamics and to potentially guide and tailor management and treatment for this group.

***Trial therapy of Intensive Short-Term Dynamic Psychotherapy (ISTDP)***. Eight musicians meeting the criteria for severe music performance anxiety (MPA) self-selected to participate in a trial treatment of ISTDP (Davanloo, 1990, 2001). All sessions were video-recorded with permission of participants and analyzed with respect to the possible etiological role that an underlying attachment disorder had on their unremitting MPA. This trial constitutes the first application internationally of ISTDP with professional musicians who had suffered severe MPA (Type 3: unresolved attachment disorder (see Kenny, 2011); also classified as fragile character structure (Davanloo, 1990, 1995).

### Hearing

A hearing health survey was conducted using a questionnaire developed for the study. This questionnaire assessed hearing conservation behaviors and the prevalence of self-perceived hearing loss. After pilot testing, it was distributed to 580 musicians across the eight participating professional orchestras. The hearing survey was administered a year after the initial physical and psychological health surveys, and took approximately 10 min for each musician to complete.

The noise exposure in practice rooms was assessed with 35 professional orchestral musicians (representing players of most orchestral instruments). These musicians were individually assessed using recording devices (Type 1 integrating sound levels meters) at each ear and in front of players as they performed a comparable pre-set practice routine in the same controlled environment.

### Surveillance

Several attempts were made to implement injury surveillance monitoring over the course of the project. Three independent approaches were trialed—one paper-based and then two web-based asking essentially the same questions. The first two were conducted within the first two and half years of the study and the third is on-going. The questions related to both playing and non-playing exposures faced by musicians, four psychological screening questions, perceived playing exertion levels [using the BORG scale (Borg, 1998)] as well as relevant outcomes (performance-related musculoskeletal disorder—PRMDs—and time lost from work) within a defined time period.

### Ethics

Ethics approval was granted by the University of Sydney Human Ethics Committee (ref: HREC1254).

## Results

From the total (*n* = 580) potential participants, 377 professional orchestral musicians (184 males, 192 females) participated in the baseline surveys (65% response rate), while a slightly higher number completed a physical assessment (70% response rate—408 in total; 199 males and 206 females (and three with unrecorded gender). More people completed the physical assessment because some people did not return fully-completed questionnaires. The mean age was 42.1 years (*SD* = 10.2). The type of instruments played by the included musicians was a good reflection of the full orchestra complement in the eight included orchestras (Table 1).

**Table 1:** **Instrument distribution in subjects included in the questionnaire and physical parts of the Sound Practice study**.

**Instrument**	**Questionnaire**		**Physical**	
	**No**.	**%**	**No**.	**%**
**UPPER STRINGS**				
Violin	115	30.5	127	31.4
Viola	54	14.3	55	13.9
Total	169	44.8	182	44.9
**LOWER STRINGS**				
Cello	46	12.2	48	11.9
Double bass	22	5.8	26	6.4
Total	68	18.0	74	18.3
**WOODWIND**				
Flute	21	5.6	22	5.4
Oboe	18	4.8	18	4.4
Bassoon	15	4.0	17	4.2
Clarinet	13	3.4	14	3.5
Total	67	17.8	71	17.5
**BRASS**				
French horn	28	7.4	28	6.9
Trombone	16	4.2	17	4.2
Trumpet	10	2.7	12	3.0
Tuba	4	1.1	4	1.0
Total	58	15.4	61	15.1
**PERCUSSION AND TYMPANI**				
Percussion	7	1.9	7	1.7
Tympani	5	1.3	6	1.5
Total	12	3.2	13	3.2
Other	3	0.8	4	1.0
Total	377	100.0	408	100.0

*Number and percent*.

### Physical

#### PRMD

Eighty four percent of the 377 participants who completed a questionnaire reported a past history of PRMDs, and 49% of the total reported PRMDs that lasted longer than a week and that were present at the time of the survey. For half of these players, PRMDs had been present for longer than 3 months. Of the 84% of musicians reporting previous performance-related injuries, on average only 40% reported full recovery from these conditions. Some of the more commonly reported pain regions, such as the shoulders, were associated with some of the worst scores in relation to recovery, averaging only 18.9% (left) and 26.4% (right) of players who reported full recovery (Ackermann et al., 2012). It is not possible to ascertain from our study whether these poor recovery rates were a consequence of inadequate rehabilitation and/or were due to the severity of the injury itself.

Injuries were most commonly reported in the spine and shoulders, but the most prevalent sites varied in relation to instrumental groups. For example, pain in the right elbow and forearm was more common for woodwind players than all other instrumental groups and typically pain was more common on the left upper limb than the right (see Table 2), despite these players having the highest scores in dexterity using the Purdue Pegboard test (Ackermann and Driscoll, 2010). The pain results by instrument group have been published previously in detail (Ackermann et al., 2012). Stratifying the results further by individual instrument played shows injury patterns that appear to be clearly related to the static and dynamic physical demands associated with playing that instrument. For example, baseline physical self-report data revealed that trombonists were significantly more likely to report left shoulder PRMDs than cellists (50% vs. 20%, *p* = 0.02), while the reverse occurred with right shoulder PRMDs, with cellists being significantly more prone to these injuries than trombonists (46% vs. 19%, *p* = 0.04). No gender effects were noted, and physical examination using a range of standard clinical tests for the shoulder did not reveal any differences related to these two instruments in terms of strength or range of motion (Driscoll and Ackermann, 2012).

**Table 2:** **Example of current pain pattern variations between instrumental groups by region in the left and right upper limbs (the right-sided scores are indicated in brackets)**.

**Region**	**Brass *n* = 58**	**Woodwind *n* = 67**	**Lower strings *n* = 68**	**Upper strings *n* = 169**	**Percussion *n* = 12**	**Total *n* = 377**
Shoulder and upper arm	10.3 (8.6)	10.4 (0)^*^	11.8 (1.5)^#^	10.1 (8.9)	16.7 (8.3)	11.1 (6.1)^#^
Elbow and forearm	3.5 (3.5)	11.9 (3)	4.4 (2.9)	6.5 (3.0)	0 (0)	6.4 (2.9)^∧^
Wrist and hand	1.7 (3.5)	1.5 (1.5)	2.9 (4.4)	4.1 (5.9)	0 (0)	2.9 (4.2)
Fingers	0 (0)	3.0 (0)	0 (1.5)	0 (1.2)	0 (0)	0.5 (0.8)
Thumb	0 (0)	4.5 (1.5)	4.4 (0)	0 (0)	8.3 (0)	1.9 (0.3)^∧^
Total	13.8	29.9	25.0	21.3	25.0	22.3

Comparison of left vs. right—

∧ p < 0.05;

# p < 0.02;

* *p < 0.01*.

#### Perceived exertion

The perceived exertion was rated as higher for performance than for rehearsing or for private practice. This was true whether the musician played in a stage or pit orchestra.

***Psychological***. All psychological screening tests for anxiety and depression (i.e., trait anxiety, social phobia, and music performance anxiety, anxiety and depression detector) were highly positively correlated; all were negatively correlated with the Core Self-Evaluation test, indicating that low self-efficacy is associated with higher psychological morbidity.

There were significant sex differences on all measures, with females consistently scoring higher than males. Younger musicians (<30) were generally more vulnerable than older musicians, particularly on the K-MPAI and SPIN. These age effects were not gender specific. On a two-item screening test for depression, 32% warranted a fuller clinical evaluation. Those who answered “yes” to both questions had significantly higher scores on the anxiety measures (STAI-T, SPIN, and K-MPAI) compared with those who answered “yes” to one or neither question. For example, those who answered “yes” to both depression questions scored a mean of 120 (*SD* = 42) on the K-MPAI. Those who answered “yes” to one depression question scored on average 92 (*SD* = 29) on the K-MPAI while those who answered “no” to both questions had a mean of 75 (*SD* = 36) on the K-MPAI.

These results indicated convergent validity for the K-MPAI with clinical tests of anxiety and depression (Kenny et al., 2012). The K-MPAI was then assessed against tests validated in clinical populations in order to develop indicative preliminary cut-off scores for use with musician populations. Receiver Operating Characteristic (ROC) Curves were generated for established clinical screening tests (STAI-T, ADD, PRIME-MD, SPIN) using different scores to dichotomize these instruments with K-MPAI as the scale measure. The cut-point for K-MPAI using Youden's Index for STAI-T ≥ 65 (1.5 SD above mean) was 105.3; using the cut point for STAI-T ≥ 60 (1 SD above mean), Youden's Index for K-MPAI was 104.5. For musicians answering yes to both depression questions on the PRIME-MD, the K-MPAI cut-point was 118.5, for one of two questions, K-MPAI cut-point was 110. For ADD, for those answering at least one question affirmatively, K-MPAI cut-point was 84.5, for those answering at least three questions affirmatively, K-MPAI cut-point was 89.5. As previously identified, K-MPAI and SPIN were unrelated (Kenny, in press).

The K-MPAI has now been translated into several languages, including French, German, Spanish, Portuguese, Spanish (continente), Portuguese (continente) Italian, Cantonese, and Polish^2^, and studies are currently occurring in these and other countries to further ascertain its convergent and discriminant validity, clinical cut points, and sensitivity and specificity for use as a clinical instrument in the population of professional musicians. Early indications are encouraging that the K-MPAI is a robust clinical instrument that may be used for diagnosis and treatment planning (see, for example, Barbar et al., 2014; Kenny, in press).

In response to a question asking musicians to rank their self-identified causes of MPA from a checklist containing 26 potential causes, the most commonly identified causes were “pressure from self,” excessive physical arousal prior to or during a performance and inadequate preparation for the performance, health issues and “tendency to be anxious in general, not just in performance” (Table 3). Fifty-five percent musicians (55%) identified that a generally high level of self-consciousness was a factor in their experience of performance anxiety. This checklist produced a very interesting pattern of results suggesting several directions for future research, including a deeper examination of the nature of the adverse experiences that exacerbated subsequent performance anxiety and how such experiences were encoded and processed by musicians.

**Table 3:** **Causes of music performance anxiety - most commonly self-identified and proportion ranked as most important**.

**Potential cause**	**Chosen (%)**	**Ranked 1 (%)**
Pressure from self	89	29
Excessive physical arousal prior to or during a performance	78	24
Inadequate preparation for the performance	63	19
Health issues	45	17
Tendency to be anxious in general, not just in performance	37	15

*Percent (n = 377)*.

Of interest, 78% musicians identified a bad performance experience as one of the causes of ongoing music performance anxiety. The relationship between self-perceived worst performance experiences and their association with subsequent self-report of MPA has also been observed in adolescent musicians (Kenny and Osborne, 2006). Music students who reported a negative music performance experience scored significantly higher on measures of MPA than those who did not report such an experience. These same students also had higher scores on trait anxiety. A bad performance experience, which constitutes a specific psychological vulnerability in terms of Barlow's (2000) triple vulnerability theory, may potentiate performance anxiety in already vulnerable musicians. This result raises the possibility that elevated MPA may develop through exposure to premature evaluative performances in a competitive environment, which is some cases, may be traumatizing. As demonstrated by in-depth interviews with professional orchestral musicians, such experiences may exert a lifelong effect, with many reporting such experiences in their formative years as a musician that have had profound effects on their subsequent management of their anxiety as adults (Kenny, 2011). Personal accounts reflected a degree of trauma resonant with post-traumatic stress. Indeed, 84 musicians (22% of the sample) responded in the affirmative with respect to a question on post-traumatic stress disorder (PTSD) on the ADD. This was an unexpected finding suggesting the need for further investigation to understand the nature of these traumas and how they might impact on music performance.

With respect to the comparative effects of different performance settings on the experience of music performance anxiety, on a 10-point Likert scale, auditions were ranked the most anxiety-provoking (mean = 8.44; *SD* = 2.07), followed by solo performance, and oral presentation, which was ranked more anxiety-provoking than a chamber music performance or orchestral concert performance.

When asked to indicate which actions they took to alleviate their music performance anxiety, musicians most commonly identified they would increase their practice, practice deep breathing, increase their positive self-talk, seek mock performance opportunities, familiarize themselves with the performance venue and take beta blockers (Table 4). Further, 5% of musicians (*n* = 17) reported drinking alcohol every day and 26.5% (*n* = 89) drank on 5–6 days per week. Alcohol frequency (i.e., drinking every day) was significantly associated with higher scores on the K-MPAI, but not on STAI-T, SPIN, or ASI. Older musicians were more likely to be daily drinkers. These results suggest that some musicians may be using alcohol to manage their performance anxiety.

**Table 4:** **Actions taken to alleviate music performance anxiety—most commonly self-identified**.

**Action taken**	**Chosen (%)**
Increase practice	59
Practice deep breathing	50
Increase positive talk	46
Seek mock performance opportunities	45
Familiarize themselves with the performance venue	42
Take beta blockers	31

*Percent (n = 377)*.

#### Associations between physical and psychological factors

Research with clinical populations has frequently observed a close relationship between reports of some forms of physical pain, in particular, chronic pain, fibromyalgia and those who frequently attend primary health care settings, with elevated psychological morbidity. This association has not been previously studied in professional musicians, despite the fact that this population experiences very high rates of performance-related musculoskeletal pain/disorder (PRMD) (Bragge et al., 2006; Ackermann et al., 2011), with various reports placing the frequency between 60 and 90% musicians. Our own study (Kenny and Ackermann, 2013) indicated that 84% professional orchestral musicians experienced PRMD, with 50% at the time of the survey reporting that they were carrying an injury/pain condition.

Accordingly, we investigated the relationship between self-reported pain conditions, an objective pain measure [trigger point pain (TPP)] and psychological morbidity (depression and anxiety) in the same population of musicians. We assessed pain frequency and pain severity using 7-point Likert scales. Only 27% musicians reported never having experienced pain related to a PRMD; 24% reported being in constant pain and 21% identified the intensity of their pain as “the worst imaginable.” There was a highly significant positive association between pain frequency and pain intensity, and between pain severity and depression. Cluster analysis using K-MPAI with severity ratings of PRMD as inputs, identified four clusters, based on the severity of PRMD scores. A significant linear association between PRMD and K-MPAI was observed—higher PRMD scores were associated with higher K-MPAI scores.

However, a more complex picture emerged for depression and PRMD pain. For three of the four clusters, the association held—higher PRMD severity—higher depression. The fourth cluster, however, comprising those who denied any depression (25% musicians), reported the highest PRMD severity (Average score = 6.65 out of a possible 7). By comparison, the group who answered “yes” to both depression questions scored an average 4.52 PRMD severity. We believe that this analysis may have identified a group of musicians who somatize their emotional distress—that is, they express their emotional pain in physical symptoms.

This is speculative and further research is needed to replicate this finding and to verify the conclusions drawn from it. Recent analyses suggest caution in over-attribution of the role of psychological factors. For example, Kenny (unpublished), using the Sound Practice database of 377 professional orchestral musicians, explored the relationship between the degree to which musicians reported recovering from performance-related physical injuries and psychological conditions such as depression, music performance anxiety and trait anxiety and found no relationship between speed or degree of recovery and these psychological factors.

Trigger point palpation (TPP in the upper trapezius muscles showed no association with PRMD severity or frequency, instead being sensitive to MPA severity. TPP ratings were highest for those reporting the highest levels of MPA and affirmative responses to both depression questions. There was a linear relationship between increasing TPP and MPA scores for the females. Men who reported the highest level of MPA, in contrast, recorded lower TPP scores than those with milder MPA. The reasons for this require further investigation.

#### Interview study

Below are examples of key themes that emerged. A more detailed account can be found in Kenny (2011).

- The struggle to separate one's musical identity from one's self-identity. One musician stated: “*Music is my life, I don't do anything else*.” The inability to derive other sources of self-worth and self-esteem leaves musicians vulnerable to feeling like failures if they fail at musical tasks such as auditions or achieving perfect performances.
- Internalized mental representations of parents which reveal the quality of attachment upon which the edifice of one's sense of self rests. Here is an example: *My father was a frustrated musician… he's an intelligent man, fantastic set of ears, a good musician but he had no formal training… He can't see anything through… he didn't give us that confidence to tackle things; we always worried that we would be failures, like him*.
- Generational transmission of music performance anxiety. This theme is related to (ii), with the addition that some musicians could make the nexus between their parents' anxiety and their own. *My mum is a real worrier; she is wound up about everything. I am like that… My mum does not seem to have come to terms with her anxiety and I have not come to terms with my anxiety… Sometimes I get nervous about the silliest things. She is like that, too*. Others were keen not to pass on their own MPA to their children.
- Adolescent onset of MPA. Many of the musicians reported sensitizing experiences during adolescence as the markers for the onset of severe MPA. *I have always suffered from performance anxiety. The first time has always stayed with me… it was related to feeling self-conscious getting up in front of hundreds of people… I thought, “WOW!” I did not know that my body could react in that way… In that performance, I really fell apart. I got really nervous and my body just let me down. It was really embarrassing. It was my first awakening to performance anxiety*.
- The stress of aging: One of our older players, a principal, was suffering very badly. We … did a temporary job swap with another player to protect him and to alleviate his stress. When players get old, you can't control things the way you used to. He was unhappy about his playing and needed to alleviate his embarrassment.
- “False alarms” (also called panic or panic attacks) appear uncued and unexpected and subsequently become conditioned in particular stressful situations that are associated with heightened threat or danger in people who have a specific psychological vulnerability and heightened neurobiological hyper reactivity. Note the description in adolescent onset of MPA, which is also an example of panic.
- Cognitive effects of anxiety impair attention and memory, and capacity to sight read.
- Situational factors: conductors, environmental challenges, work issues, lack of peer support (critical, competitive environment).

#### Trial of ISTDP

ISTDP's theoretical rationale draws on attachment theory (Bowlby, 1960, 1973), whose core therapeutic action is the “patient's actual experience of their true feelings about the present and the past” (Davanloo, 1990) (p. 2). Although psychodynamic in theoretical structure, the main areas of innovation of ISTDP lie in its therapeutic practices. Davanloo (1990, 2001, 2005) developed a technique to rapidly mobilize the unconscious therapeutic alliance, called the central dynamic sequence (Davanloo, 1987) in order to remove the major resistances to change, which are not effectively removed through interpretation alone. To date, one successful case study has been published (Kenny et al., 2014) and others are in preparation.

### Hearing

#### Usage hearing protection devices

All orchestras in the study group were aware of the risks to their musicians and were actively taking some steps to reduce noise exposure, with varying degrees of effectiveness and understanding of the issues as reported by the authors previously (O'Brien et al., 2012). The reported regular use of adequate personal hearing protection by musicians was 64%. Newly developed hearing protection devices incorporating active, level-dependent technology were found to be more acceptable to orchestral musicians who trialed these devices than any previous personal protective device, with results described in detail elsewhere (O'Brien et al., 2014).

#### Exposure to noise

During practice, sound levels were recorded at between 60 and 107 dB LA_eq_, with peak levels between 101 and 130 dB LC_peak_. For average reported practice durations (2.1 h per day, 5 days a week), 53% would exceed accepted permissible daily noise exposure in solitary practice, in addition to sound exposure during orchestral rehearsals and performances. Similar to levels experienced in the orchestra, those most at risk of NIHL while practicing were the brass and percussion, with significant differences noted in sound levels recorded between ears in the violin, viola, flute/piccolo, horn, trombone, and tuba. Full details are described in the *Journal of the Acoustical Society of America* article (O'Brien et al., 2013a).

### Surveillance

Following discussions with orchestra management and musicians, a paper-based system was implemented with the intention of musicians completing the two-page form on a regular basis before or after scheduled rehearsals, as it was expected this would maximize participation. Several important issues were identified during the trial implementation. These included allocating time for the form completion around the orchestra rehearsal schedule; balancing the frequency of data collection with the proportion of musicians who completed the forms; the level of detail of information collected; the areas covered by the data collection; the format of the form; and the musicians' perceptions of the usefulness of the information collected.

Subsequent to this first trial, a majority of musicians requested a web-based system. There was a range of opinions regarding the optimum frequency for data collection (weekly, fortnightly or four-weekly). Therefore, the first web-based system allowed the musician to choose the frequency of data entry. Uptake was better with the original web-based system but still low. The main issues identified were difficulties maintaining regular email contact (due to changing email addresses and firewall issues); developing a workable system of reminders; making the entry of data on anatomical site and symptoms simple; and maintaining interest amongst the musicians. The key area of improvements were determined to be the need for automatic regular feedback to musicians regarding the data they had entered previously and the ability to extract data easily for analysis purposes. A new surveillance system, developed in cooperation with the (Galbraith, 2014) which has an operating model for dance, is now being developed and will be trialed in 2014. This new version of the on-line system is menu-driven as much as possible, but it will be possible to enter text in some areas. Regular reminders are included and feedback is provided to allow the individual to compare their exposure data with others who play the same instrument and overall.

## Discussion

The *Sound Practice* program was designed to provide a detailed understanding of OHS aspects of being a professional orchestral musician in Australia, and to trial a series of interventions aimed to improve health and well-being in these musicians. To date, the study has provided a great deal of useful information on the complex health issues faced by professional orchestral musicians and on interventions that may be of benefit. A multi-disciplinary approach has confirmed the interactive relationship between factors such as psychological and physical well-being, and reinforces the need for a holistic approach to better preventing and managing health issues arising in the orchestral workplace.

### Physical

Orchestral musicians in Australia report a high rate of occupational injury, consistent with reports from overseas. There has been little change in these statistics over several decades (Fry, 1986; Fishbein et al., 1988). This may be related to little information being available on normal physical characteristics of performers, as well as a lack of evidence for effective intervention strategies. In addition, the loading induced by the instrument itself appeared to influence the site and nature of injury. Specific increased demands producing static postural loading (e.g., in the left shoulder of trombonists) and/or dynamic movement challenges (e.g., in the right shoulder of cellists) are thought to be the likely reason for this effect.

This study has provided normative values for musicians for many physical attributes (size, strength, and range of movement) of musicians and lends some support to the notion that musicians show some adaptive musculoskeletal changes in response to playing demands, such as the increased supination seen in the left forearm of violin and viola players (Ackermann and Driscoll, 2010; Ackermann et al., 2012).

The assessment protocol used a wide range of traditional physical tests that had been shown to be among the best available in terms of their sensitivity, specificity, and clinical utility in a usual clinical environment (Ackermann and Driscoll, 2010). However, these proved to be limited in assessing musicians' injuries. According to the measurements obtained, musicians, if anything, seem to show physical attributes above the normal benchmarks, as may be expected in an elite or “hyperfunctioning” population. As an example, the differences seen between the shoulder PRMDs in cellists and trombonists with distinctly different performance postural and biomechanical demands, and not identified in the standard clinical shoulder physical examination protocols, highlights the need for more specific clinical assessment approaches for musicians.

### Psychological

In this study, the K-MPAI proved to be sensitive to age and sex differences and had significant correlations with most of the other anxiety measures, providing early evidence for the convergent validity of K-MPAI. The results highlight the need for broader psychological testing in the assessment of musicians presenting with problematic MPA, in particular, tests of PTSD and depression. Medication use should also be evaluated, given the high use in this population. Patient education of other psychological approaches should be included in management strategies.

As well as showing the extent of underlying psychological difficulties, this study also indicates that a complex relationship exists between psychological and physical wellbeing in this population. Professional orchestral musicians attending healthcare clinics for pain or injury conditions should be screened for psychological problems as attempts to treat the physical pain may not be effective unless the psychological issues are addressed simultaneously. Our findings provide some support for previous work (Leaver et al., 2011) indicating the need to be aware of possible somatization, and to continue with research to clarify the role of somatization in musicians whose injuries are not responsive to physical therapies. Screening tests will alert treating practitioners to the possible presence of psychological conditions that may need to be managed concurrently with the physical injury.

We strongly caution against over-attribution of psychological causes to physical injuries; each musician must be assessed individually to obtain a complete profile of their psychological and physical needs. The physical aspects of physical injuries must remain center stage in the treatment plan because a physical injury is often just that!

### Hearing

The results show that musicians are at risk of NIHL while playing in the orchestra, but also face significant additional exposure during solitary practice, compounding their risk (O'Brien et al., 2012, 2013a). As has been previously recommended, the uptake and use of earplugs by musicians is unpopular (Laitinen and Poulsen, 2008; Zander et al., 2008) but needs to be increased to reduce the potential risk of NIHL (Emmerich et al., 2008; Jansen et al., 2009; Pawlaczyk-Łuszczyńska et al., 2010; Toppila et al., 2011). In Australia, while legislation has enhanced the use of earplugs and awareness of hearing issues, the findings in this study concur with the reports of hearing loss and dysfunction in orchestral musicians reported in these other international studies. Such data reinforces the need to continue to find better solutions to noise exposure as technology continues to develop in this field (O'Brien et al., 2013b, 2014). Focusing on educating musicians and orchestral management on best-practice hearing protection strategies is also important.

### Surveillance

Useful surveillance is difficult to establish and maintain; must be developed taking particular account of the needs, interests, and attitudes of musicians; and is probably most likely to be effective with inclusion of regular and timely feedback to participants of their own results and the broader findings of the surveillance program.

## Conclusions

The professional orchestral musician population remains susceptible to a range of workplace health and safety risks, but exposures vary between instruments and orchestras. Some specific areas that need to be addressed by further research include: refining models of musculoskeletal examination to include instrument-specific evaluation; ensuring that the psychological well-being of a musician is evaluated in conjunction with any chronic or major playing-related health conditions; continuing to explore effective ways of protecting hearing without damaging the ultimate performance goal of making beautiful music; and further trial and modification of an on-line approach to musicians surveillance.

### Conflict of interest statement

The authors declare that the research was conducted in the absence of any commercial or financial relationships that could be construed as a potential conflict of interest.

## Footnotes

^1^Questionnaires developed for Sound Practice project and included in baseline questionnaire. See also Kenny et al. (2012) for detailed descriptions.

^2^Certified translations of the K-MPAI in the languages specified above are available upon request from the author (dianna.kenny@sydney.edu.au).
